# Cardioprotective Activity of Pharmacological Agents Affecting NO Production and Bioavailability in the Early Postnatal Period after Intrauterine Hypoxia in Rats

**DOI:** 10.3390/biomedicines11102854

**Published:** 2023-10-21

**Authors:** Olena Popazova, Igor Belenichev, Nina Bukhtiyarova, Victor Ryzhenko, Valentyn Oksenych, Aleksandr Kamyshnyi

**Affiliations:** 1 Department of Histology, Cytology and Embryology, Zaporizhzhia State Medical and Pharmaceutical University, 69000 Zaporizhzhia, Ukraine; 2 Department of Pharmacology and Medical Formulation with Course of Normal Physiology, Zaporizhzhia State Medical and Pharmaceutical University, 69000 Zaporizhzhia, Ukraine; 3 Department of Clinical Laboratory Diagnostics, Zaporizhzhia State Medical and Pharmaceutical University, 69000 Zaporizhzhia, Ukraine; 4 Department of Medical and Pharmaceutical Informatics and Advanced Technologies, Zaporizhzhia State Medical University, 69000 Zaporizhzhia, Ukraine; 5 Broegelmann Research Laboratory, Department of Clinical Science, University of Bergen, 5020 Bergen, Norway; 6 Department of Microbiology, Virology and Immunology, I. Horbachevsky Ternopil State Medical University, 46001 Ternopil, Ukraine

**Keywords:** prenatal hypoxia, cardioprotective, heat shock proteins, Angiolin, L-arginine, Thiotriazoline, Mildronate, eNOS, iNOS, nitrotyrosine

## Abstract

Intrauterine hypoxia in newborns leads to a multifaceted array of alterations that exert a detrimental impact on the cardiovascular system. The aim of this research was to assess the cardioprotective effects of modulators of the nitric oxide (NO) system, including L-arginine, Thiotriazoline, Angiolin, and Mildronate, during the early postnatal period following intrauterine hypoxia. Methods: The study involved 50 female white rats. Pregnant female rats were given a daily intraperitoneal dose of 50 mg/kg of sodium nitrite starting on the 16th day of pregnancy. A control group of pregnant rats received saline instead. The resulting offspring were divided into the following groups: Group 1—intact rats; Group 2—rat pups subjected to prenatal hypoxia (PH) and daily treated with physiological saline; and Groups 3 to 6—rat pups exposed to prenatal hypoxia and treated daily from the 1st to the 30th day after birth. Nitrotyrosine levels, eNOS, iNOS, and NO metabolites were evaluated using ELISA; to measure the expression levels of iNOS mRNA and eNOS mRNA, a PCR test was utilized. Results: Angiolin enhances the expression of eNOS mRNA and boosts eNOS activity in the myocardium of rats with ischemic conditions. Arginine and particularly Thiotriazoline exhibited a consistent impact in restoring normal parameters of the cardiac nitroxidergic system following PH. Mildronate notably raised iNOS mRNA levels and notably reduced nitrotyrosine levels, providing further support for its antioxidative characteristics.

## 1. Introduction

Despite remarkable strides in both fundamental and clinical medicine, alongside advancements in the efficacy of perinatal care services, the prevalence of neonates who have encountered intrauterine hypoxia persists at unaltered rates. Moreover, in certain instances, there exists an alarming inclination towards its escalation. Intrauterine hypoxia experienced by newborns triggers a complex array of changes that exert an unfavorable impact on the central nervous system (CNS) and its regulatory influence on organs and systems, particularly the cardiovascular system [1]. At the core of cardiovascular disturbances following intrauterine hypoxia are disruptions in the mechanisms of neural and humoral regulation of heart and vascular function, as well as abnormalities in the conduction system (sinus tachy- and bradycardias, Hiss bundle branch blockade), transient neonatal pulmonary hypertension, prolonged persistence of fetal circulatory pathways, and impairment of cardiomyocyte metabolism [2]. Post-hypoxic cardiopathy serves as a risk factor for the development of cardiovascular pathologies (rhythm disorders, vascular dystonia, etc.) in subsequent age periods [3].

According to modern understanding, endothelial dysfunction and related disruptions in the NO system contribute to the development of numerous cardiovascular diseases. It is well-documented that fluctuations in NO levels in the body during pregnancy can result in classic symptoms of eclampsia, alterations in placental development, modifications in placental blood circulation, embryo- and fetopathies, intrauterine growth retardation, and fetal mortality [4]. NO may serve as a factor in both the pathogenesis and as a cyto- and organ-protective agent, particularly in terms of cardiovascular and endothelial protection [5,6]. Under prenatal hypoxic conditions, NO production increases, but concurrently, the synthesis of essential factors responsible for NO storage and transport decreases, resulting in NO deficiency in the heart and blood vessels. In prolonged prenatal hypoxia, reactive oxygen species (ROS) may impact the bioavailability of NO [7]. The increase in ROS during prenatal hypoxia and the disruption of the ROS/NO balance in the fetus result in heightened peripheral vasoconstriction, potentially leading to hypoxia in vital organs such as the heart and brain [8]. Prenatal hypoxia (PH) increases the expression of NADPH 1 homologue, which stimulates the production of superoxide. Superoxide can subsequently react with NO to form the stable peroxynitrite anion, thereby reducing the bioavailability of NO [9].

Hence, contemporary therapy for chronic fetoplacental insufficiency has incorporated L-arginine-based preparations (as a source for NO synthesis) and medications aimed at enhancing the bioavailability of NO [10,11]. Several studies have demonstrated the cardioprotective and endothelioprotective qualities of medications capable of enhancing both NO synthesis and its bioavailability. L-arginine, Thiotriazoline, Angiolin, and Mildronate are noteworthy in this context. L-arginine serves as a substrate for NO production in vascular endothelial cells, exhibiting antioxidant, cytoprotective, anti-hypoxic, and membrane-stabilizing attributes, and has been utilized in the management of gestosis and fetoplacental insufficiency [12].

Thiotriazoline (morpholinium 3-methyl-1,2,4-triazolyl-5-thioacetate) is a specific scavenger of NO and its cytotoxic forms. It increases NO bioavailability, protecting it from ROS. Thiotriazoline is also known for its anti-ischemic properties; it enhances the compensatory activation of the malate-aspartate shuttle mechanism, reduces the inhibition of oxidation processes in the Krebs cycle, and preserves intracellular ATP reserves. Thiotriazoline is employed in obstetric practice as part of a comprehensive approach in cases of threatened miscarriage [13].

Angiolin, also known as L-lysine 3-methyl-1,2,4-triazolyl-5-thioacetate, enhances the expression of VEGF and the density of proliferating endothelial cells within muscle-type vessels and microcirculatory channels. It boosts NO availability, elevates eNOS expression, and reduces the levels of peroxidation products during myocardial ischemia. Angiolin exhibits cardioprotective attributes in cases of myocardial ischemia, preserving mitochondrial ultrastructure and elevating ATP levels in the myocardium through the activation of the compensatory malate-aspartate shuttle mechanism [14].

Mildronate inhibits the conversion of gamma-butyrobetaine, a precursor of carnitine, which leads to the accumulation of gamma-butyrobetaine. This accumulation stimulates NO synthesis and has a positive impact on the endothelium of cardiac vessels [15]. Mildronate decreases the transport of long-chain fatty acids across mitochondrial membranes, which are mediated by carnitine, while it does not influence the metabolism of short-chain fatty acids. It also activates an alternative energy production system, involving glucose oxidation, and demonstrates anti-ischemic and cardioprotective characteristics [16]. Mildronate demonstrates anti-hypoxic properties in experimental cases of fetoplacental insufficiency [17].

Thus, the search for and development of approaches to pharmacotherapy targeting prenatal myocardial injury through modulation of the NO system is a pertinent task in contemporary pharmacology. The aforementioned rationale theoretically supports the potential of studying NO system modulators with diverse mechanisms of action—L-arginine, Thiotriazoline, Angiolin, and Mildronate as means of cardioprotection of posthypoxic cardiovascular system disorders in newborns.

The purpose of this study was to evaluate the cardioprotective activity of NO system modulators (L-arginine, Thiotriazoline, Angiolin, and Mildronate) in the early postnatal period after intrauterine hypoxia.

## 2. Materials and Methods

### 2.1. Description of Laboratory Animals and the PH Experimental Model

The research involved 50 female outbred white Wistar rats and 10 males, with weights ranging from 220 to 240 g and an age of approximately 4.5 months. These rats were procured from the vivarium of the Institute of Pharmacology and Toxicology at the National Medical Academy of Ukraine. They were housed in standard vivarium conditions, with temperatures maintained between 20 °C and 25 °C, humidity levels at 50–55%, and natural lighting; they were provided with a diet appropriate for this laboratory animal species and had access to water without restrictions. In this study, we utilized a model inducing chronic hematitic nitrite-induced prenatal hypoxia (PH) [14,18]. In order to establish a fixed pregnancy period, mature male rats were placed with virgin female rats with a ratio of 2 males per 4 females. The pregnancy timeline commenced upon the detection of spermatozoa in the vaginal smear (designated as day 1 of pregnancy). To induce hematitic hypoxia during the prenatal phase of development, pregnant female rats were subjected to daily intraperitoneal injections of sodium nitrite solution from day 16 to day 21 of pregnancy, at a dosage of 50 mg/kg, which induced a state of moderate hypoxia [18]. Pregnant rats in the control group were administered a physiological solution under the same conditions. The offspring was categorized into the following groups: group 1 consisted of intact, healthy rat pups born to females with physiologically normal pregnancies who were administered a physiological solution; group 2 encompassed pups from the control born after PH and daily administered a physiological solution; groups 3 to 6 consisted of pups after PH that received daily drugs treatments from postnatal day 1 to day 30. A portion of the rats was removed from the experiment on 30 days immediately after the end of administration of pharmacological agents, and a portion of the rats was removed 60 days after birth (30 days after administration of pharmacological agents)

The doses of L-arginine and Mildronate were taken from open literature sources. The doses of Thiotriazoline and Angiolin were calculated experimentally; these data are presented in the preclinical research report.

### 2.2. Justification for the Selected Drugs and Their Characteristics

We opted for medications with established experimental evidence demonstrating their capacity to regulate the nitric oxide (NO) system:

1. The intact group consisted of rats born from mothers with normal pregnancies, and they were administered a physiological solution.
2. The control group comprised rats born after experiencing intrauterine hypoxia, and they were given a physiological solution.
3. Thiotriazoline (Morpholinium-3-methyl-1,2,4- triazolyl-5-thioacetic acid) (2.5% solution for injections, “Arterium”, Kyiv, Ukraine), metabolitotropic cardioprotector and antioxidant, 50 mg/kg, i/p [19].
4. Angiolin ([S]-2,6-diaminohexane acid 3-methyl-1,2,4-triazolyl-5-thioacecate) (substance, research, and production association “Farmatron”, Zaporizhzhia, Ukraine), anti-ischemic, endothelium protective drug, 50 mg/kg, i/p [20].
5. L-arginine (42% solution for injection in vial, Tivortin, Yuria-pharm, Cherkasy, Ukraine), an NO precursor; it mitigates disruptions in the nitroxidergic system in ischemia, 200 mg/kg, i/p [21].
6. Mildronate (2-(2-carboxyethyl)-1,1,1-trimethylhydrazinium) (10% injection solution in ampoules, Grindex (Riga, Latvia), metabolitotropic agent, 100 mg/kg, i/p [22].

### 2.3. Anaesthesia

Under the administration of thiopental anesthesia (40 mg/kg), rats from all experimental groups were taken out of the study. Following this, blood samples were obtained from the celiac artery for subsequent analysis.

### 2.4. Preparation of Biological Material

The heart was rinsed with chilled 0.15 M KCl (4 °C) in a 1:10 ratio. After removing excess fat, connective tissue, and cutting out blood vessels and clots from internal cavities, the heart was washed again with 0.15 M KCl (4 °C) in a 1:10 ratio. Subsequently, it was pulverized in liquid nitrogen to achieve a powdery consistency. On a WT500 torsion balance (manufactured in Moscow, Russia), 100 mg of heart tissue, previously ground into a fine powder using liquid nitrogen, was accurately weighed. The powdered tissue was then thoroughly mixed with 10.0 mL of a medium maintained at 2 °C. This medium contained the following components in millimoles per liter (mmol/L): sucrose (250 mmol/L), Tris-HCl buffer (20 mmol/L), and EDTA (1 mmol/L), adjusted to a pH of 7.4. The homogenate was subsequently subjected to a pre-centrifugation step using a Sigma 3-30k refrigerated centrifuge (Osterode am Harz, Germany) at (+4 °C) for 7 min at 1000× *g* to remove large cell fragments. The resulting supernatant was carefully collected and then subjected to a second centrifugation step at (+4 °C) for 20 min at 17,000× *g* using the same Sigma 3-30k refrigerated centrifuge (Germany). The supernatant obtained from this step was collected and stored at −80 °C. The dense mitochondrial precipitate obtained after resuspension was utilized for further investigations. The determination of nitrotyrosine, iNOS, eNOS, and the concentration of NO metabolites (NOx) was carried out on the resuspended fraction.

The apex of the heart was placed in Bouin’s fixative for 24 h. Following the standard process of tissue dehydration, and chloroform and paraffin impregnation, the myocardium was embedded in paraplast (McCormick, Hunt Valley, MD, USA). Serial histological sections, 5 μm thick, were prepared on a Microm-325 rotary microtome (Microm Corp., Munich, Germany). Subsequent to treatment with 0-xylene and ethanol, these sections were used for real-time PCR analysis.

### 2.5. Enzyme-Linked Immunosorbent Assay (ELISA)

Nitrotyrosine was assessed in the cytosol homogenate of the heart using the solid-phase immunoassay sandwich method of ELISA. The ELISA Kit (Catalog No. HK 501-02) from Hycult Biotech, Uden, Netherlands was used according to the instructions.

The activity of endothelial nitric oxide synthase (eNOS) in the cytosol was determined through enzyme immunoassay using the Cloud-Clone Corporation kit (Katy, TX, USA, #PAA868Ra01), according to the instructions.

The activity of inducible nitric oxide synthase (iNOS) in the cytosol was assessed through enzyme immunoassay using the MyBioSource kit (San Diego, CA, USA, #MBS023874), according to the instructions. These analyses were conducted on a complete plate enzyme immunoassay analyzer (SIRIO-S, Seac, Italy).

### 2.6. Biochemical Methods

The levels of NO metabolites (NOx) in the hearts were assessed using the Griess method. The supernatant obtained as above (1.0 mL) was deproteinized by adding 100 µL of 0.092 M zinc sulfate and 100 µL of 1 M NaOH, stirred, and left for 30–40 min. Then, it was centrifuged at 4000× *g* for 10 min (at 5 °C) using an Eppendorf™ 5430 G centrifuge (Hamburg, Germany). Subsequently, 100 µL of the resulting supernatant was transferred to a well in a microplate, and 0.5 mM of vanadium (III) chloride was added to each well to reduce nitrate to nitrite. Following this, 50 μm of sulfonamide and 0.2 μm of *N*-1-(naphthyl) ethylenediamine were added. The total volume of the incubation mixture is 300 µL. The next step was to incubate the samples for 30 min at 37 °C, and the optical density was measured at 540 nm. The concentration of NOx was determined using a linear standard curve within the range of 0–50 μmol/L sodium nitrate. NOx levels in the tissues were expressed in μmol/L.

### 2.7. Reverse Transcription Real-Time Polymerase Chain Reaction (RT-PCR)

Total RNA was extracted from the heart samples using a standard protocol using NucleoZOL (Macherey-Nagel, Düren, Germany). The extracted RNA was dissolved in RNase-free water to obtain a concentration of 2 µg/µL. The weight of the heart fragments was 30–80 mg. cDNA (complementary DNA) synthesis was performed using a RevertAid First Strand cDNA Synthesis Kit (Thermo Fisher Scientific, Waltham, MA, USA; K1621) according to the manufacturer’s instructions. We homogenized tissue samples with a rotor-stator homogenizer for mechanical disruption using up to 100 mg of tissue per 1 mL NucleoZOL (Macherey-Nagel™, Düren, Germany). For tissues with high DNA content, it is recommended to use 50 mg of tissue/mL reagent. To process the sample in 1.5 mL or 2 mL microcentrifuge tubes, we used an 880 μL aliquot of the homogenate (80 mg tissue + 800 μL NucleoZOL). Residual homogenate can be stored at −20 °C or −70 °C for at least one year for later use.

Real-time RT-PCR (Polymerase Chain Reaction) amplification was performed to quantify the expression levels of iNOS and eNOS mRNA, using Maxima SYBR Green/ROX qPCR Master Mix (2×) (ThermoScientific, Waltham, MA, USA) with gene-specific primers on a Biorad CFX 96 Real-Time PCR Detection System. Specific primer pairs β-actin (5′-3′) (Forward primer = ACAACCTTCTTGCAGCTCCTC; Reverse primer. = TCGTCATCCATGGCGAACTGG), iNOS (5′-3′) (Forward primer = GTTCCTCAGGCTTGGGTCTT; Reverse primer. = CCGTGGGGCTTGTAGTTGAC), and eNOS (5′-3′) (Forward primer = CCCAGGAGAGATCCACCTCA; Reverse primer. = CAGCACATCCTGGGTTCTGT) for the analysis of both the target and reference genes were meticulously selected using the PrimerBlast tool (www.ncbi.nlm.nih.gov/tools/primer-blast, accessed on 1 June 2023) and procured from ThermoScientific, USA. The reaction mixture contained 10 µL of 2 × Maxima SYBR Green/ROX qPCR Master Mix, 0.5 µL of each gene-specific primer, 2 µL of cDNA template, and nuclease-free water to a final volume of 20 µL. The PCR cycling conditions involved initial denaturation at 95 °C for 10 min, followed by 45 cycles of denaturation at 95 °C for 15 s, primer annealing at 60 °C for 40 s, and elongation at 72 °C for 40 s. The registration of fluorescence intensity occurred automatically at the end of the elongation stage of each cycle through the automatic SybrGreen channel.

The actin beta (Actb) gene was used as the reference gene to normalize the expression levels of the target genes. The expression levels of the target genes were quantified relative to the expression of the housekeeping gene using the comparative Ct (2^−ΔΔCt^) method. The Ct values were converted to relative expression values using the formula 2^−ΔCt^, where ΔCt = (Ct target gene − Ct housekeeping gene). The relative expression values were then converted to Log2 values using the formula Log2 (relative expression).

### 2.8. Statistical Analysis

Statistical analyses were conducted using “STATISTICA^®^ for Windows” (StatSOFT, Hamburg, Germany). Group comparisons were assessed using either one-way ANOVA or ANOVA for repeated measurements, followed by post hoc Bonferroni correction or the Kruskal–Wallis criterion with subsequent Dunn correction. Statistical significance was determined at a threshold of *p* < 0.05.

## 3. Results

Modeling of prenatal hypoxia (PH) leads to persistent impairments of the nitroxidergic system of the heart in offspring at 1 and 2 months of age. Thus, in the hearts of 1-month-old rats after PH, a 55% reduction in eNOS compared to healthy animals of the same age group was observed. Additionally, animals in this group exhibited a 2-fold increase in iNOS compared to healthy animals (Table 1). We also found an elevation in the mRNA expression of iNOS in the hearts of 1-month-old rats after PS by 7.2 times and an 85% decrease in eNOS mRNA compared to the group of healthy 1-month-old animals (see Table 2). A significant decrease of 49.2% in NO metabolites was observed in the hearts of 1-month-old animals after PH, in comparison to the group of 1-month-old rats born to mothers with normal pregnancies. This reduction suggests a decrease in NO production by endothelial nitric oxide synthase (eNOS), with the NO generated through the activation of iNOS expression being converted into peroxynitrite. The oxidative conversion of NO is evidenced by a 4.5-fold increase in nitrotyrosine concentration compared to the group of healthy rats (Table 1). These findings indicate substantial disruptions in the myocardial NO system of rats after PH, including alterations in NOS expression patterns, reduced NO bioavailability, and the activation of nitrosative stress.

Similar changes in the nitroxidergic system of the heart persisted 2 months after PH (Table 3 and Table 4). Thus. in the heart of 2-month-old rats after PH, a decrease in eNOS by 54.8% and in eNOS mRNA by 77% was registered in comparison with the group of healthy 2-month-old rats. Higher values of iNOS, by 2-fold, and iNOS mRNA, by 6.9-fold, compared to the group of healthy animals were also observed. In 2-month-old rats, a myocardial deficiency of stable NO metabolites was observed, with a significant decrease of 38.8% compared to intact values. Nitrotyrosine concentration in the heart of 2-month-old rats after PH remained high and exceeded the values of healthy 2-month-old rats by 3.4 times.

The course of administration of pharmacological preparations having properties of NO modulators during the 30-day period immediately after birth to post-PH rats influenced the indices of cardiac nitroxidergic system (Table 1, Table 2, Table 3 and Table 4). Thus, immediately after discontinuation of the experimental therapy, the best indices were in the group receiving Angiolin. Angiolin administration leads to an increase in eNOS by 2.9 times and in eNOS mRNA by 32 times, and a decrease in iNOS by 50.4% and in iNOS mRNA by 3.8 times, in the heart of 1-month-old rats after PH compared to the group of 1-month-old rats after PH without treatment (control). The administration of Angiolin led to a notable 60% reduction in nitrotyrosine levels in the rat myocardium following prenatal hypoxia. Additionally, there was a substantial 81.2% increase in stable NO metabolites. This observation suggests that Angiolin effectively enhances both the production and bioavailability of NO. Administration of Thiotriazoline had a favorable effect on the state of the nitroxidergic system of the heart of 1-month-old PH rats. Thus, in this group immediately after the course administration of Thiotriazoline, there was an increase observed in eNOS by 50%, in eNOS mRNA by 15 times, and in stable metabolites of NO by 62.5%, and a decrease in iNOS by 22.5%, in iNOS mRNA by 73.8%, and in nitrotyrosine by 35%, in comparison with the control group. It can be concluded that Thiotriazoline, as well as its structural analog Angiolin, is able to increase NO production and its bioavailability. The effects of arginine on the indices of the nitroxidergic system of the heart of 1-month-old rats after PH were comparable to those of the group receiving Thiotriazoline. Immediately after course administration of meldonium to post-PH rats, no significant changes in nitrotyrosine concentration and eNOS mRNA expression were detected, and iNOS mRNA expression in the heart of this group was significantly higher than that in the control group.

The studies performed in the heart of 2-month-old animals after PH showed a persistent therapeutic effect after the use of NO modulators. Thus, as in the first observation. Angiolin application remained the most effective. In the heart of 2-month-old rats 1 month after 30-day Angiolin administration, eNOS increased by 2.85 times and eNOS mRNA by 23 times compared to control values, and iNOS decreased by 50% and iNOS mRNA by 81.5% compared to control values. Administration of Angiolin resulted in a 56.8% increase in stable NO metabolites.

The level of nitrotyrosine in the heart of this group did not differ significantly from that in the group of healthy 2-month-old rats. Arginine and, especially, Thiotriazoline also demonstrated a persistent effect on the normalization of the nitroxidergic system of the heart after PH. At 1 month after a course administration of Thiotriazoline, higher values compared with the control (eNOS by 50% and eNOS mRNA by 12 times) and lower values compared with the control (iNOS by 41.5% and iNOS mRNA by 56%) were observed. In this group, the concentration in the heart of stable NO metabolites was 47.7% higher, and the nitrotyrosine concentration in the heart was lower than that in the control group by 41.7%. One month after the course administration of meldonium, the indices of the nitroxidergic system did not differ from the control values. The only difference was a decrease in the level of nitrotyrosine in the heart of 2-month-old rats after PH.

**Figure 1:**
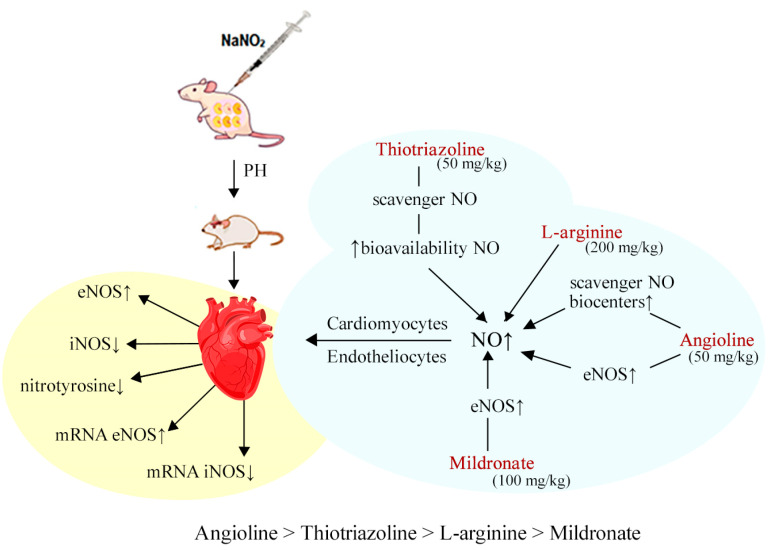
The cardiovascular system indicators in both the blood and heart of rats subjected to prenatal hypoxia and subsequent pharmacological modulation of the NO system.

Thus, we have established a positive effect of Angiolin, Thiotriazoline, and arginine on the indices of the nitroxidergic system of the heart of rats after PH, both at the end of the 30-day experimental treatment and one month after its cancellation (Figure 1).

## 4. Discussion

**Figure 2:**
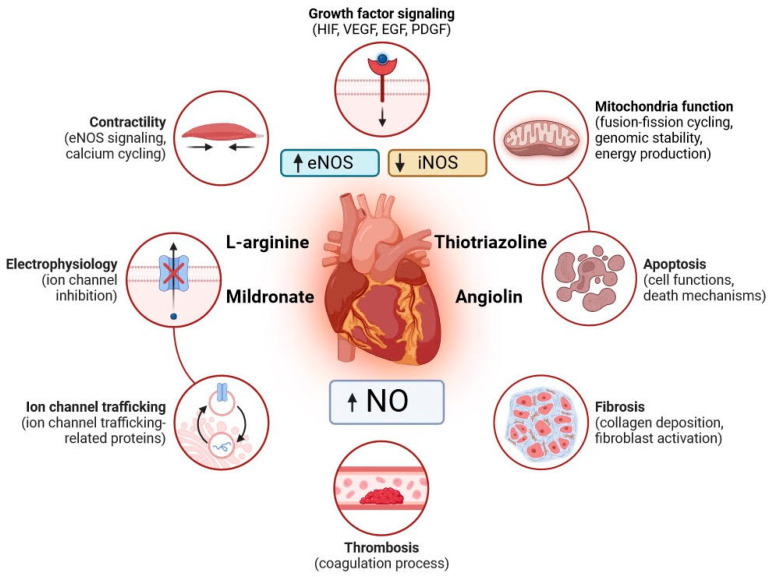
Indicators of the cardiovascular system in the blood and heart of rats subjected to prenatal hypoxia and subsequent pharmacological modulation.

The revealed persistent disturbances of the nitroxidergic system of the heart after experimental PH consist of the suppression of eNOS expression by the increased expression of its inducible form, decreased NO bioavailability, and activation of nitrosative stress as evidenced by increased nitrotyrosine. (Figure 2). The disorders revealed by us are in line with modern views on the mechanisms of myocardial damage during ischemia and hypoxia, which have been established through experimental research and clinical observations [23,24]. PH is known to impair cardiac tolerance to ischemia/reperfusion, damage endothelium-dependent vasodilation/vasoconstriction mechanisms, and further contribute to cardiovascular pathologies such as hypertension, atherosclerotic vascular disease, and congestive heart failure [25]. There is evidence for decreased eNOS expression and activity in cadiomyocytes and endothelium and a risk of endothelial dysfunction after intrauterine hypoxia. Disruption of eNOS activity can be explained by altered interactions between eNOS and its regulatory partner proteins such as caveolin-1, calmodulin, and Hsp90. Alterations in phosphorylation and dephosphorylation of key serine and threonine residues in eNOS may also be involved in impaired eNOS activity [26,27]. Disturbances of endothelium-dependent vasodilation in coronary arteries of male and female offspring who underwent PH at the age of 4 and 9.5 months against the background of decreased eNOS and impaired function of SKCa and IKC channels were revealed [28]. Low eNOS values lead to impaired NO-dependent regulation of glutathione synthesis and reduced resistance to oxidative stress [29]. Decreased eNOS may be due to HIF-1a deficiency, as this factor activates eNOS expression by phosphorylation of a serine residue [30]. Increased iNOS expression was detected during the eNOS decrease after PH, which is aimed at compensating NO production [31]. High iNOS activity may be associated with cofactor deficiency and the release of superoxide and other reactive forms of NO [32].

However, under conditions of reduced thiol antioxidant deficiency, this leads to the formation of cytotoxic NO derivatives in “parasitic” reactions. Other researchers found that in preeclampsia, low activity of endothelial synthesis of NO and redox-dependent transformation of NO to peroxynitrite leads to a decrease in blood NO [5]. Such reactions can occur under conditions of L-arginine deficiency, antioxidant deficiency, mitochondrial dysfunction, and increased iNOS expression. Uncontrolled formation of cytotoxic derivatives of NO leads to nitrosylation of the most active parts of protein structures of ion channels, receptors, transmembrane pores, and signaling molecules, i.e., development of nitrosative stress.

A less important consequence of myocardial ischemia is the loss of such NO-mediated effects as suppression of cell proliferation and platelet aggregation, and, most importantly, inhibition of monocyte activation by the so-called adhesion molecules [33]. Nitrosative stress also leads to HSP70 deficiency in the cell, together with deprivation of the glutathione link of the thiol-disulfide system. Cytotoxic forms of NO lead not only to modification (reversible and irreversible) of macromolecules, including HSP70 itself, but also reduce the expression activity of genes encoding the synthesis of the latter [34,35]. The role of NO derivatives in suppressing gene activity and reducing the levels of various transcription factors has been demonstrated [36,37]. Apparently, excess forms of nitric oxide, such as peroxynitrite and nitrosonium ions, first nitrolyse the thiol–redox-dependent regions of these genes, then, at increasing concentrations, oxidize them [38,39]. All this justifies the use of pharmacological agents—modulators of the NO system—for myocardial protection after PH.

L-arginine is a common substrate for NO and polyamines (putrescine, spermine, and spermidine). NO and polyamines play an important role in reproduction, embryogenesis, reduction in neonatal mortality, and embryonic angiogenesis. NO regulates gene expression, protein synthesis, proliferation, growth, and differentiation of the fetus [40]. Therefore, the gaze of researchers and clinicians has been turned to the use of the NO substrate L-arginine to reduce the adverse effects of PH [12]. Our studies revealed a positive effect of arginine on NO system parameters in the heart of 1- and 2-month-old rats after PH.

However, the NO substrate was inferior to new molecules—Thiotriazoline and Angiolin. We speculate that in ischemia, NO formed from arginine under thiol antioxidant deficiency interacts with ROS and is converted to peroxynitrite [41]. In this regard, pharmacological agents combining the properties of positive modulators of NO and its transporters are promising.

Thiotriazoline is able to increase NO bioavailability in excess ROS. Thiotriazoline is an antioxidant and ROS scavenger, and NO increases the activity of glutathione-dependent enzymes and the level of reduced glutathione during myocardial ischemia. Thiotriazoline (10-5–10-7 M) in vitro reduced the levels of superoxide radicals and peroxynitrite due to the presence of a thiol group in its structure. Thiotriazoline prevents irreversible inactivation of the transcription factor NF-kB, protecting sensitive cysteine residues—Cys 252, Cys 154, and Cys 61 in its DNA-binding domains—from excess ROS. Thiotriazoline can participate in the restoration of these groups in the case of reversible inactivation, taking on the role of Redox Faktor-1. Thiotriazoline enhances the activation of expression of redox-sensitive genes that are essential for cellular defense against oxidative stress. Thiotriazoline reduces the intensity of nitrosative stress and increases eNOS activity [42,43].

Thiotriazoline increases the efficacy of arginine when co-administered. The pharmacological effect of the combination is due to a positive effect on the synthesis, transport, and bioavailability of NO and physiological functions of this molecular messenger [44]. In rats in which isadrin-pituitrin myocardial infarction was modeled, Thiotriazoline stimulated LDH in the direction of the formation of pyruvate from lactate, which eliminated lactic acidosis and normalized intracellular pH, and stimulated the Krebs cycle by increasing pyruvate [45]. In the same experimental mode, Thiotriazoline activated the malate-aspartate shunt in the myocardium in the acute period of myocardial infarction. Thiotriazoline administration (600 mg/day) to 8298 patients with II–III class stable angina pectoris reduced the number of weekly angina attacks by 46.32%, and in the control group by 33.24% (*p* = 0.028), respectively (*p* = 0.031), and increased exercise tolerance [46].

Experimental studies have established that Angiolin increases eNOS mRNA expression and eNOS activity in ischemic rat myocardium. Angiolin increases VEGF expression and the VEGF binding coefficient with vascular endothelium, as well as the density of endotheliocytes and proliferating endotheliocytes of the capillary network and vascular wall, and increases RNA concentration in endotheliocytes during hypoxia and circulatory ischemia [14]. Angiolin has a protective effect on NO and also increases its bioavailability; NO is an unstable, short-lived radical, and to prolong its “life”, the formation of more stable S-nitrosol complexes with low molecular weight compounds (glutathione, cysteine) is envisaged, and with a deficiency of these compounds, NO bioavailability sharply decreases. With a deficiency of low-molecular thiols, NO under the action of ROS is transformed into peroxynitrite and can be the cause of initiation of nitrosative stress. Due to its chemical structure, Angiolin plays the role of a spin trap and can form a complex with NO [47]. Angiolin positively affects the state of myocardial nitroxidergic system in experimental ischemia—it increases NO synthesis by increasing eNOS expression, increases NO bioavailability, and reduces parasitic reactions by decreasing iNOS hyperactivity. The mechanism of the effect on eNOS expression can be explained in terms of Angiolin’s effect on HSP70 and HIF-1a. Angiolin prolongs the “life time” of HIF-1a through HSP70-mechanisms. Angiolin also has a positive effect on the glutathione link thiol-disulfide system, which is conjugated with the NO system. It was revealed that in experimental myocardial ischemia, Angiolin increases the activity of glutathioreductase and glutathione peroxidase, and increases the concentration of reduced glutathione in the cytosol of rat myocardium [35]. HIF-1a is also known to increase eNOS and VEGF expression during hypoxia and ischemia [48]. Angiolin, due to its positive effect on the NO system, positively influenced cardio- and hemodynamics in experimental myocardial ischemia. Angiolin administration to rabbits with occlusion of the descending coronary artery led to restoration of left ventricular dysfunction, which was expressed in the increase in the left ventricular working index and left ventricular working stroke index, the increase in pressure in the left ventricle, and the decrease in total peripheral vascular resistance [14].

Mildronate (3-(2.2.2.2-trimethylhydrazine) propionate) reversibly blocks gamma-butyrobetaine hydroxylase, which catalyzes the conversion of gamma-butyrobetaine to carnitine, and thereby significantly inhibits the entry of carnitine, which provides transport of fatty acids across the membrane into muscle tissue cells. This effect of Mildronate is accompanied by a decrease in carnitine-dependent oxidation of free fatty acids (FFAs) and, consequently, leads to activation of glucose oxidation, which is more economical in conditions of ischemia. An important feature of the action of Mildronate, which distinguishes it from other drugs affecting myocardial metabolism, is the absence of accumulation of under-oxidized fatty acids inside mitochondria and an increase in NO production. This is due to the fact that Mildronate inhibits the hydroxylation of GBB and increases the intracellular pool of γ-butyrobetaine, whose esterification exhibits cholinomimetic properties. γ-butyrobetaine esters via acetylcholine receptors on endothelial cells can activate NOS [41]. Mildronate improves exercise tolerance and quality of life of patients, and positively affects the functional parameters of the heart-ejection fraction and systolic volume in myocardial ischemia [15]. Our studies confirmed the anti-hypoxic activity of Mildronate in PH. However, in this study, we did not find a significant positive effect of Mildronate on NO system parameters in the myocardium of animals undergoing PH. It was found that Mildronate significantly increased iNOS mRNA expression and significantly decreased nitrotyrosine concentration, which is more evidence of its antioxidative properties [49].

## 5. Conclusions

We observed that intrauterine hypoxia suppressed eNOS mRNA expression and eNOS activity, while increasing iNOS mRNA expression and iNOS activity in the myocardium of offspring at 1 and 2 months of age. Furthermore, this hypoxic condition resulted in reduced NO bioavailability and the activation of nitrosative stress in the neonatal myocardium. The results of our experimental study underscore the essential need for pharmacological interventions to correct myocardial NO system disorders in newborns. This represents a critical component of early and necessary cardioprotective measures following intrauterine hypoxia. While examining agents with reported NO-modulating properties, we discovered that not all of them had a positive impact on the myocardial NO system in newborns following experimental prenatal hypoxia (PH). Our study revealed that Thiotriazoline and Angiolin exhibited significant benefits by enhancing NO synthesis, improving bioavailability, and reducing nitrosative stress in the newborns’ myocardium after PH. Our studies support further preclinical investigation of Angiolin as a potential cardioprotective agent following experimental prenatal hypoxia (PH). Additionally, the results indicate the potential for further preclinical and clinical research on Thiotriazoline, an authorized drug, as a treatment for cardiovascular pathologies resulting from intrauterine hypoxia.

## Figures and Tables

**Table 1:** Effect of treatment on nitroxidergic system indicators in the cytosolic fraction of homogenates from 1-month-old rats after prenatal hypoxia and treatment.

Experimental Groups	eNOS, pg/mL	iNOS, pg/mL	Nitrotyrosine, pg/mL	NO Metabolites (NOx), μmol/L
Intact (Rats born from rats with normal pregnancies) (*n* = 10)	22.4 ± 1.77	13.1 ± 1.36	12.1 ± 1.10	6.3 ± 0.54
PH (Rats with prenatal hypoxia) (control) (*n* = 10)	10.1 ± 1.44 ^1^	26.7 ± 2.55 ^1^	54.2 ± 4.12 ^1^	3.2 ± 0.43 ^1^
PH +L-arginine (*n* = 10)	18.4 ± 1.45 *	24.1 ± 4.55 ^1,^*	44.5 ± 3.41 ^1,^*	4.6 ± 0.53 *
PH + Thiotriazoline (*n* = 10)	15.2 ± 1.75 ^1,^*	20.7 ± 2.55 ^1,^*	35.3 ± 2.61 ^1,^*	5.2 ± 0.35 *
PH + Angiolin (*n* = 10)	29.2 ± 2.76 ^1,^*	17.7 ± 1.35 ^1,^*	22.3 ± 1.42 ^1,^*	5.8 ± 0.54 *
PH + Meldonium (*n* = 10)	12.4 ± 2.45 ^1^	28.8 ± 2.44 ^1^	52.7 ± 4.34 ^1^	3.7 ± 0.47

Notes: ^1^—*p* ≤ 0.05 in relation to the intact group of animals; *—*p* ≤ 0.05 in relation to the control group of animals.

**Table 2:** Effect of treatment on eNOS and iNOS mRNA expression in myocardial tissues of 1-month-old rats after prenatal hypoxia.

Experimental Groups	mRNA eNOS. u.e.	mRNA iNOS. u.e.
Intact (Rats born from rats with normal pregnancies) (*n* = 10)	1.000 ± 0.0032	1.000 ± 0.011
PH (Rats with prenatal hypoxia) (control) (*n* = 10)	0.15 ± 0.00019 ^1^	7.22 ± 0.0313 ^1^
PH +L-arginine (*n* = 10)	0.790 ± 0.00011 ^1,^*	4.82 ± 0.0045 ^1,^*
PH + Thiotriazoline (*n* = 10)	2.295 ± 0.0093 ^1,^*	3.88 ± 0.0017 ^1,^*
PH + Angiolin (*n* = 10)	4.876 ± 0.0214 ^1,^*	1.89 ± 0.016 ^1,^*
PH + Meldonium (*n* = 10)	0.164 ± 0.0017 ^1^	8.11 ± 0.0061 ^1,^*

Notes: ^1^—*p* ≤ 0.05 in relation to the intact group of animals; *—*p* ≤ 0.05 in relation to the control group of animals.

**Table 3:** Effect of treatment on nitroxidergic system parameters in the cytosolic fraction of 2-month-old rat homogenates after prenatal hypoxia and treatment.

Experimental Groups	eNOS, pg/mL	iNOS, pg/mL	Nitrotyrosine, pg/mL	NO Metabolites (NOx), μmol/L
Intact (Rats born from rats with normal pregnancies) (*n* = 10)	31.2 ± 2.65	16.1 ± 1.42	14.1 ± 1.31	7.2 ± 0.67
PH (Rats with prenatal hypoxia) (control) (*n* = 10)	14.1 ± 1.54 ^1^	33.7 ± 3.74 ^1^	48.3 ± 3.88 ^1^	4.4 ± 0.35 ^1^
PH +L-arginine (*n* = 10)	26.2 ± 2.22 ^1,^*	29.1 ± 2.32 ^1^	40.2 ± 2.67 ^1,^*	5.8 ± 0.44 *
PH + Thiotriazoline (*n* = 10)	21.2 ± 1.8 ^1,^*	19.7 ± 2.23 *	28.1 ± 1.44 ^1,^*	6.5 ± 0.57 *
PH + Angiolin (*n* = 10)	40.2 ± 3.65 ^1,^*	16.7 ± 1.47 *	18.0 ± 2.05 *	6.9 ± 0.72 *
PH + Meldonium (*n* = 10)	18.4 ± 2.22 ^1^	27.8 ± 4.77 ^1^	40.3 ± 3.32 ^1,^*	4.8 ± 0.53

Notes: ^1^—*p* ≤ 0.05 in relation to the intact group of animals; *—*p* ≤ 0.05 in relation to the control group of animals.

**Table 4:** Expression of eNOS mRNA and iNOS mRNA in myocardial tissues of 2-month-old rats after prenatal hypoxia and treatment.

Experimental Groups	mRNA eNOS. u.e.	mRNA iNOS. u.e.
Intact (Rats born from rats with normal pregnancies) (*n* = 10)	1.000 ± 0.0078	1.000 ± 0.0076
PH (Rats with prenatal hypoxia) (control) (*n* = 10)	0.23 ± 0.0002 ^1^	6.89 ± 0.0093 ^1^
PH +L-arginine (*n* = 10)	0.718 ± 0.0002 ^1,^*	3.90 ± 0.0022 ^1,^*
PH + Thiotriazoline (*n* = 10)	2.770 ± 0.0113 ^1,^*	3.011 ± 0.0021^1,^*
PH + Angiolin (*n* = 10)	5.410 ± 0.037 ^1,^*	1.272 ± 0.0081^1,^*
PH + Meldonium (*n* = 10)	0.27 ± 0.0082 ^1^	6.7103 ± 0.009 ^1^

Notes: ^1^—*p* ≤ 0.05 in relation to the intact group of animals; *—*p* ≤ 0.05 in relation to the control group of animals.

## Data Availability

All the data generated during this research are included in the manuscript.
